# The origin and succession of the microbial community in decomposing litter

**DOI:** 10.1093/ismeco/ycaf155

**Published:** 2025-09-05

**Authors:** Jiancheng Chen, Edith Bai, Yuting Liang, Ziping Liu, Yaxin Ji, Tongtong Sun, Zhenxin Guo, Yingdong Huo, Shasha Liu, Björn Berg

**Affiliations:** Key Laboratory of Geographical Processes and Ecological Security of Changbai Mountains, Ministry of Education, Changchun, Jilin, 130024 China; School of Geographical Sciences, Northeast Normal University, Changchun, Jilin, 130024 China; Key Laboratory of Geographical Processes and Ecological Security of Changbai Mountains, Ministry of Education, Changchun, Jilin, 130024 China; School of Geographical Sciences, Northeast Normal University, Changchun, Jilin, 130024 China; Key Laboratory of Vegetation Ecology, Ministry of Education, Northeast Normal University, Changchun, Jilin, 130024 China; State Key Laboratory of Soil and Sustainable Agriculture, Institute of Soil Science, Chinese Academy of Sciences, Nanjing, Jiangsu, 210008 China; University of Chinese Academy of Sciences, Beijing, 100049 China; University of Chinese Academy of Sciences, Nanjing, Jiangsu, 211135 China; Key Laboratory of Geographical Processes and Ecological Security of Changbai Mountains, Ministry of Education, Changchun, Jilin, 130024 China; School of Geographical Sciences, Northeast Normal University, Changchun, Jilin, 130024 China; Key Laboratory of Geographical Processes and Ecological Security of Changbai Mountains, Ministry of Education, Changchun, Jilin, 130024 China; School of Geographical Sciences, Northeast Normal University, Changchun, Jilin, 130024 China; Key Laboratory of Geographical Processes and Ecological Security of Changbai Mountains, Ministry of Education, Changchun, Jilin, 130024 China; School of Geographical Sciences, Northeast Normal University, Changchun, Jilin, 130024 China; Key Laboratory of Geographical Processes and Ecological Security of Changbai Mountains, Ministry of Education, Changchun, Jilin, 130024 China; School of Geographical Sciences, Northeast Normal University, Changchun, Jilin, 130024 China; Key Laboratory of Geographical Processes and Ecological Security of Changbai Mountains, Ministry of Education, Changchun, Jilin, 130024 China; School of Geographical Sciences, Northeast Normal University, Changchun, Jilin, 130024 China; Key Laboratory of Geographical Processes and Ecological Security of Changbai Mountains, Ministry of Education, Changchun, Jilin, 130024 China; School of Geographical Sciences, Northeast Normal University, Changchun, Jilin, 130024 China; Department of Forest Sciences, University of Helsinki, 00014 Helsinki, Finland

**Keywords:** decomposing litter, microbial origin, assembly mechanisms, niche

## Abstract

Litter decomposition is an important process of nutrient cycling and is primarily driven by microbes. However, whether the microorganisms in decomposing litter come from the phyllosphere or soil is still unclear. In this study, we collected litter of two dominant species in a temperate forest, *Fraxinus mandshurica* and *Pinus koraiensis* (newly shed, and decomposing litter in the early-stage and late-stage) and surrounding soil to approach this question. Our results suggested that in the early-stage of decomposition, phyllosphere bacteria utilized readily available substances, preempting the niche of soil bacteria, while soil fungi were able to invade the litter through hyphae and spores. These activities further modified the ecological niche in the decomposing litter, facilitating the subsequent entry of soil bacteria and fungi. The timing of soil microbial invasion was influenced by litter quality. In the low-quality litter, the resource limitation hindered the entry of soil microorganisms and consequently slowed down the decomposition process. These findings offer crucial insights for better understanding of the litter decomposition process during which substantial carbon is lost from the ecosystem.

## Introduction

Throughout the life cycle of plants, microorganisms coexist with plants both on the surface and in the interior zones of plant tissues, together forming a meta-community [1, 2]. These microorganisms not only exist on the living plants but also play an important role in the litter decomposition process. Litter decomposition is an essential ecological process that contributes to the formation of soil carbon pool [3–5], which is mainly affected by climate conditions, litter quality [6, 7], and decomposers such as soil fauna [8] and microbes [9]. The microorganisms involved in litter decomposition are important, but whether they originate from the phyllosphere, soil, or air is still unclear.

Phyllosphere, the aboveground part of plant, harbors microbes in both epiphytic and endophytic niches [10]. They are already present in the newly shed plant litter and could affect the subsequent succession of microbial communities in the decomposing litter via niche modification (nutrients and quality of litter is modified by phyllosphere microbes for faster decomposition by soil microbes) and niche pre-emption (colonization by soil bacteria and fungi is impaired by the resident phyllosphere community) [11, 12]. Although these two mechanisms are totally contrasting, with niche modification suggesting more favorable niches while niche pre-emption suggesting less favorable niches for soil microbes created by phyllosphere microbes, they both suggested the importance of phyllosphere microbes for the “Home-field advantage” (HFA, plants often associate with specialized decomposer communities that enhance plant litter breakdown at their home-field) effects. Niche modification mechanism suggests the modified niche by phyllosphere microbes is more suitable for soil microbial decomposition of litter at “home” than “foreign” environment, while niche pre-emption mechanism suggests “home” environment favors the persistence of phyllosphere endophytes that specialize in decomposing recalcitrant carbon more than “foreign” environment [13]. At present, we do not know which mechanism dominated the HFA effect or the succession process of microbes during litter decomposition. In addition, because the existing phyllosphere community and soil generalists were both found to display non-negligible decomposition abilities [13–15], understanding whether the decomposers in litter originate mainly from soil or phyllosphere microbes can help us to better understand not only the mechanisms of HFA, but also the dynamics of carbon and nutrient cycling during litter decomposition.

In the limited number of studies on the shift from phyllosphere to soil communities during litter decomposition, researchers have found the importance of phyllosphere fungi, showing similar fungal taxa in decomposing *Quercus petraea* litter to those in living leaves [16] and a more significant role of fungi than bacteria as decomposition proceeds [17]. These studies of microbial succession have not been linked to the studies of sources of microbes in decomposing litter and a more comprehensive exploration of both the phyllosphere (both epiphytic and endophytic) and soil microorganisms is needed.

In this study, epiphytic and endophytic microbes in litter from different stages of decomposition for two plant species *F. mandshurica* (FM, ash) and *P. koraiensis* (PK, pine) from different altitudes in Changbai mountains were studied. Newly shed litter that had not yet touched the ground, the early-stage decomposing litter (partly decomposed for ~9 months with their species still being identifiable), and the late-stage decomposing litter (decomposed for ~21 months) and soil samples were collected. We hypothesized that: (i) Soil microbes would contribute more than phyllosphere microbes in the decomposing litter due to the higher amount of microbial biomass in soils than in the phyllosphere; (ii) whether niche pre-emption or niche modification prevails would be dependent on the ability of soil microbes. For example, soil fungi have higher ability to pass environmental limitations through hyphae or spores, therefore being less affected by niche pre-emption and more affected by niche modification than soil bacteria; (iii) Litter quality would also affect whether niche pre-emption or niche modification prevails. Pine litter would have a more stable (more resistant) ecological niche throughout the decomposition process than ash litter due to its lower available resources [18], such as space, energy or nutrients, facilitating the preservation of phyllosphere microbes. The knowledge of this study can provide a scientific foundation for better understanding of microbial succession and litter decomposition and better preservation of biodiversity.

## Materials and methods

### Experimental site and sampling

This study was conducted in Changbai Mountains, Jilin Province, China, focusing on two dominant tree species in a broad-leaved mixed forest: *F. mandshurica* (FM, ash, and high quality litter) and *P. koraiensis* (PK, pine, and low quality litter). Our research sites spanned four different altitudes: 750 m (42°24′ N, 128°5′ E), 800 m (42°20′ N, 128°5′ E), 900 m (42°18′ N, 128°6′ E), and 1050 m (42°14′ N, 128°8′ E). At each altitude, four trees of each species were selected, which had similar age and growth rate; all eight trees fell within a 30 × 30 meter plot. Newly shed litter that had not yet touched the ground was collected. Surrounding soil was also collected together with decomposing litter. Additionally, a portion of litter, designated as late-stage decomposing litter, was placed into litter bags and retrieved the following year together with the surrounding soil, but it should be noted that at the 900 m site the ash litter was almost completely decomposed and therefore no samples were obtained. For both ash and pine litter, early and late-stage decomposing litter were defined as litter decomposed for 9 months and 21 months, respectively. Ash litter had undergone around 50% decomposition and was still identifiable but had lost structural integrity at the sampling time of as early-stage decomposition. Only a little amount of ash litter was left for analysis after a year. Pine litter at the sampling time of early-stage decomposition was darkish and partially decomposed with identifiable morphology. After a year it turned entirely black and structurally loose. We are aware that these calendar-based stages may not represent equivalent biological decomposition states due to inherent differences in decomposition rates between species.

### Chemical properties analyses

Litter samples were dried to a constant mass at 105°C and then ball-milled to prepare for the analysis of total carbon and nitrogen using an elemental analyzer. Cellulose and lignin content of litter was measured by Van Soest method [19]. Enzyme activity was measured by the method described previously [20].

### Sequencing

DNA was extracted according to the method described in Zhu *et al.* [21]. In the early-stage decomposing litter, considering that the ecological niche of litter was still unstable, we separated the surface and internal microorganisms of the litter. For the litter surface samples, ~8–10 g of litters were transferred into a 250 ml conical flask. The bottles were shaken at 180 rpm and 30°C on a shaking incubator for 1 h after adding 100 ml of 0.01 M, phosphate-buffered saline (pH = 7.4). Phosphate buffer was initially filtered through a nylon gauze followed by filtration through a 0.22 μm cellulose membrane. For interior microbial analysis, litter samples were submerged in 30% H_2_O_2_ for 30 min to remove the surface microbial community. This was followed by three times of washing with sterilized water. The samples were then treated with 70% ethanol for 1 min and rinsed again in sterilized water. To confirm that the surface disinfection process was successful, the last wash solution for each sample was incubated in tryptic soy broth (TSB) culture, at 37°C, 180 rpm, for 7 days, to confirm that there were no phyllosphere microorganisms remaining [21]. DNA extraction was subsequently carried out on both the cellulose membrane and litters. For late-stage decomposing litter, we extracted DNA directly.

The quality and concentration of DNA were determined by 1.0% agarose gel electrophoresis and a NanoDrop2000 spectrophotometer (Thermo Scientific, United States) and kept at −80°C prior to further use. Bacterial 16S rRNA amplicon libraries were constructed through a two-step PCR process. Initially, we employed primers 799F (5′–AACMGGATTAGATACCCKG–3′) and 1392R (5′–ACGGGCGGTGTGTRC–3′) for the first PCR amplification round. PCR conditions were 95°C for 3 min, followed by 27 cycles of denaturation at 95°C for 30 s, annealing at 55°C for 30 s, and extension at 72°C for 45 s. The process was completed with a final extension at 72°C for 10 min and then maintained at 10°C until manually halted. Subsequently, a second PCR amplification was carried out on the purified amplicons, utilizing primers 799F (5′– AACMGGATTAGATACCCKG–3′) and 1193R (5′–ACGTCATCCCCACCTTCC–3′), employing a similar thermal profile but with an adjusted cycle count of 13 cycles. The hypervariable region ITS1 of the fungal ITS gene were amplified with primer pairs ITS1F (5′ – CTTGGTCATTTAGAGGAAGTAA–3′) and ITS2R (5′ – GCTGCGTTCTTCATCGATGC–3′). PCR conditions were 95°C for 3 min; followed by amplification for: 35 cycles of 30 s at 95°C, 35 s at 55°C and 45 s at 72°C. These primers were chosen specifically to minimize the amplification of plant chloroplast and mitochondrial genes, a critical consideration for phyllosphere samples rich in host plant DNA [22].

Purified amplicons were pooled in equimolar amounts and paired-end sequenced (2 × 300 bp) on an Illumina MiSeq platform (Illumina, San Diego, CA, United States) according to standard protocols by Majorbio Bio-Pharm Technology Co. Ltd. (Shanghai, China).

### Processing of sequencing data

Our analysis employed the DADA2 [23] plugin within QIIME2 (2021.2) [24] for denoising purposes. The first step involved filtering out low-quality sequences to ensure the reliability of our dataset. Based on quality scores, both 16S and ITS reads were truncated and quality filtered. Truncation lengths were chosen when the 25th percentile quality score fell below Q30. The pipeline accounted for the natural biological length variation of the ITS1 region without requiring fixed-length trimming. Then, nontarget sequences assigned as mitochondrial or chloroplast origin were filtered from the dataset by QIIME taxa filter-table Plugins. DADA2 denoised sequences are usually called amplicon sequence variants (ASVs), forming the basis of our study. With a refined dataset, we proceeded to construct an evolutionary tree to conduct diversity analysis. Phylogenetic analysis was performed by aligning representative ASVs using MAFFT, masking hypervariable regions, and constructing an approximately maximum likelihood phylogenetic tree using FastTree. Taxonomic assignment was conducted using a Naive Bayes classifier trained on reference sequences from the Greengenes2 [25] database for bacterial 16S rRNA and the UNITE v8.3 [26] database for fungal ITS regions. In total, we had 4 448 624 rarified 16S sequence reads and 9 125 133 ITS sequence reads, which resulted in 42 525 bacterial ASVs and 30 158 fungal ASVs, respectively.

### Statistical analysis

As there was no significant variation in microbial diversity across altitudes (Table S1), we considered samples from different altitudes as replicates in our subsequent analyses. However, they were not merged in the source-sink analysis, as microbes from different altitudes cannot directly interact. We conducted a one-way analysis of variance to investigate the differences in the litter properties of *F. mandshurica* and *P. koraiensis* in newly shed, early and late stages decomposing litter. Subsequently, we utilized QIIME2 to calculate alpha and beta diversity. The Shannon index was employed to quantify alpha diversity, and we applied the Kruskal-Wallis H test for intra-group comparisons. Beta diversity was represented by the unweighted UniFrac distance. To statistically assess the significance of group differences, we conducted pairwise permutational multivariate analysis of variance (PERMANOVA) using the pairwise.adonis function. This method performed pairwise comparisons between all groups, calculating *P-*values through permutation tests to determine whether observed differences were statistically significant. The results indicated the proportion of variance explained by each group and provide adjusted *P-*values to control for multiple testing.

To track the potential microbial origins, we used the Fast expectation–maximization microbial source tracking (FEAST) [27], which employs the expectation–maximization (EM) algorithm to iteratively estimate the contributions of microbial sources to a given sink community. The model operates under the assumption that the sink is a convex combination of all known sources, supplemented by an uncharacterized “unknown” source to account for unexplained variation. During the Expectation (E) step, the algorithm calculates the posterior probabilities that each taxon originates from each source. During the Maximization (M) step, it updates the weights of source contributions and refines their abundance profiles, simultaneously allocating any residual unexplained composition to the “unknown” source. This iterative framework supports efficient and scalable analysis of thousands of potential sources while maintaining a balance between computational efficiency and the resolution of complex microbial community structures. We performed co-occurrence network analysis through ggClusterNet [28], selecting the top 400 abundant ASVs with a correlation coefficient (r.threshold) >0.8 and a significance level (p.threshold) <0.01. Gephi 0.10.1 [29] was used for visual representation of these networks. To evaluate the importance of each species in the network, we employed a leave-one-out approach [30, 31]. Each species was sequentially removed, and the mean values of degree centrality, betweenness centrality, and closeness centrality were recalculated for the remaining network. The impact score of each species was defined as the sum of the absolute changes in these centrality measures. Species with impact scores in the top 10% were identified as keystone species. And we calculated niche overlap by focusing on keystone species exclusively identified within the phyllosphere or soil origin of the early-stage decomposing litter network. Subsequently, the role of stochastic and deterministic processes in the assembly of microbial communities was evaluated using the phylogenetic-bin-based null model analysis (iCAMP) [32]. Specifically, iCAMP was conducted in two separate analyses: one including early-stage litter, phyllosphere, soil, and the other including late-stage litter, phyllosphere, and soil. This design allowed us to evaluate community assembly processes within each decomposition stage, and to compare across the three habitat types (litter, phyllosphere, and soil), rather than across stages directly. For the visual interpretation of these findings, Interactive Tree of Life (iTOL) was employed [33, 34]. Except for the FEAST (version 3.6.3) analysis, all data processing tasks were carried out using R version 4.2.3.

## Results

### Source of microbial communities involved in the decomposing litter

**Figure 1:**
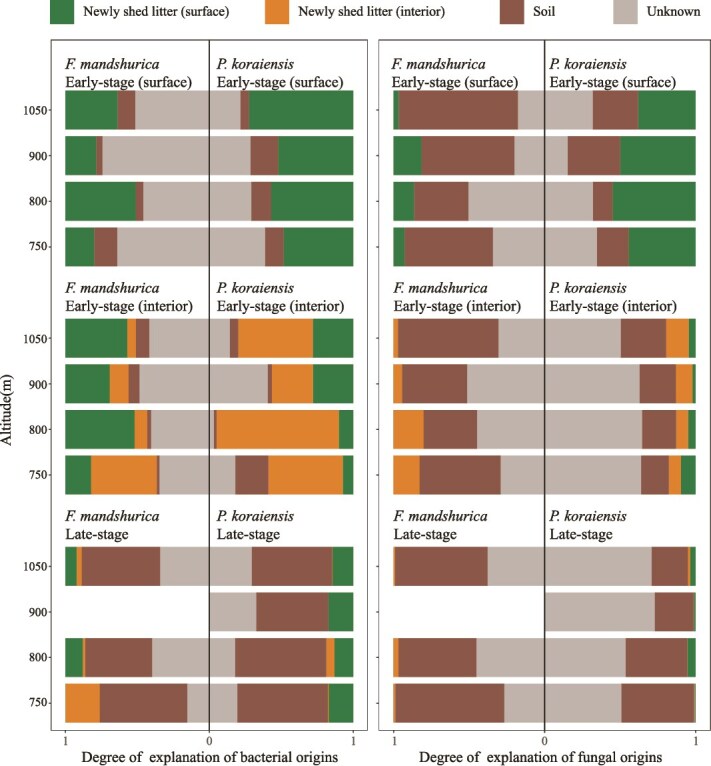
The origins of bacteria (left) and fungi (right) in decomposing litter of two plant species along the altitude gradient. Origins were detected for the surface and interior microorganisms of the early-stage and late-stage decomposing litter of *F. Mandshurica* and *P. Koraiensis.* The degree of explanation was calculated by the fast expectation–maximization microbial source tracking (FEAST).

For surface bacteria involved in the early-stage decomposing litter (Fig. 1), the phyllosphere bacteria (here represented by the newly shed litter) were the main source for both ash (20.2%–49.0%) and pine (48.5%–72.4%) litter, while soil contributed 4.3%–16.0% and 6.0%–19.0%. For interior bacteria (Fig. 1), the phyllosphere bacteria were the main source in the early-stage decomposing litter of ash (44.0%–63.5%) and pine (56.6%–95.1%). Contrastingly, the contribution of soil bacteria with 46.6%–60.9% in ash litter and 50.0%–63.4% in pine litter to the late-stage decomposing litter was higher than that of phyllosphere bacteria (11.5%–24.0% in ash litter and 14.9%–18.7% in pine litter, Fig. 1). In the decomposing early-stage ash litter, a larger proportion of fungi were traced back to soil sources than to phyllosphere sources. Specifically, soil sources accounted for 36.0–79.0% on the surface and 35.4–66.7% in the interior, whereas phyllosphere sources contributed only 3.4–18.6% on the surface and 5.7–19.9% in the interior (Fig. 1). On the surface of the early-stage decomposing pine litter, the soil contribution (13.0–34.7%) was lower than that of the phyllosphere (38.2–55.1%). However, the primary fungal source for the interior of pine litter was unknown. In the decomposing late-stage litter, fungi from the phyllosphere were nearly absent, moreover, sources of fungi in pine litter largely remained unknown (Fig. 1).

**Figure 2:**
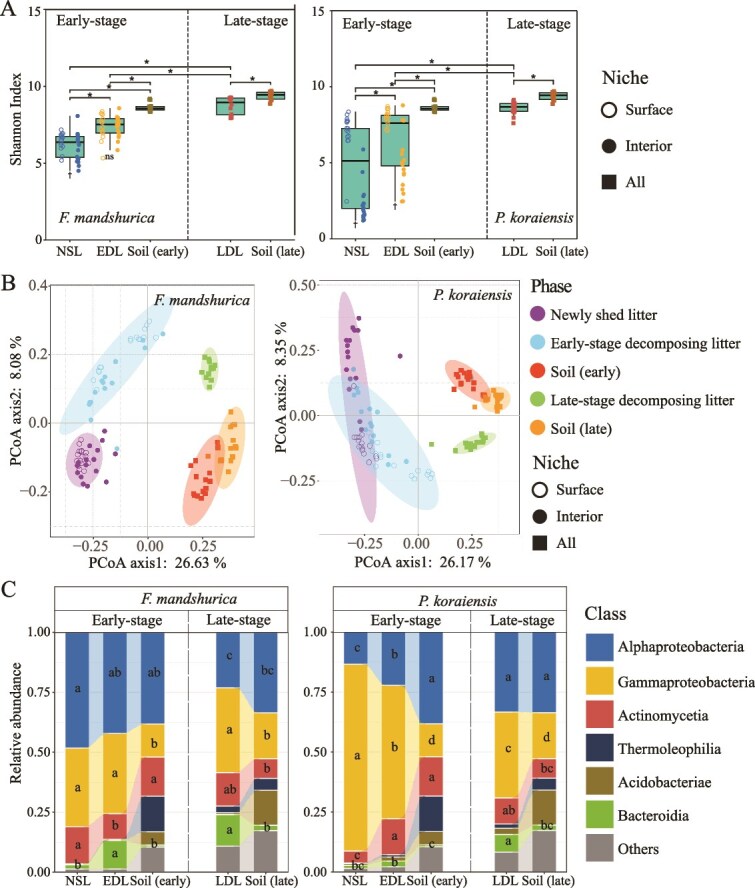
Diversity and species composition of the bacterial community in the phyllosphere, early-stage and late-stage decomposing litter. (A): Shannon index of bacteria in the newly shed litter (NSL), early-stage decomposing litter (EDL), late-stage decomposing litter (LDL) and soil for *F. Mandshurica* and *P. Koraiensis*. The “^*^” represents a significant difference (*P* < .05) between groups, and the “†” represents a significant difference (*P* < .05) between the surface and interior bacteria during that phase. ns: non-significant. (B): Principal-coordinate analysis (PCoA) plots of the bacterial community (using unweighted UniFrac method). (C): Relative abundance of the bacteria in the NSL, EDL, LDL, and the soil. Different letters indicate a significant difference (*P* < .05).

**Figure 3:**
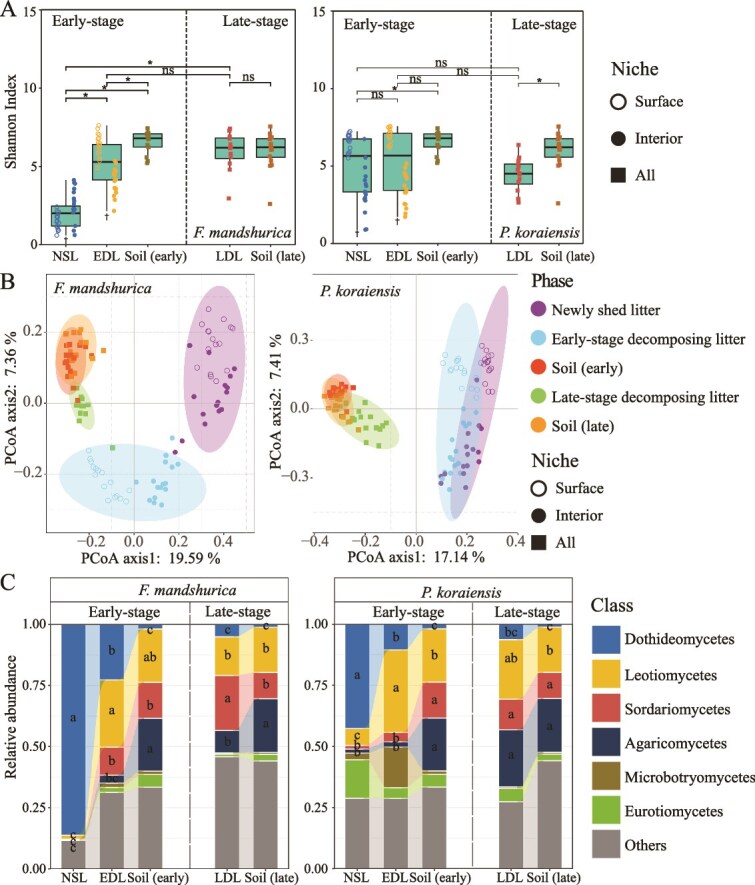
Diversity and species composition of the fungal community in the phyllosphere, early-stage and late-stage decomposing litter. (A): Shannon index of fungi in the newly shed litter (NSL), early-stage decomposing litter (EDL), late-stage decomposing litter (LDL) and soil for *F. Mandshurica* and *P. Koraiensis*. The “^*^” represents a significant difference (*P* < .05) between groups, and the “†” represents a significant difference (*P* < .05) between the surface and interior fungi during that phase. ns: non-significant. (B): Principal-coordinate analysis (PCoA) plots of the fungal community (using unweighted UniFrac method). (C): Relative abundance of the NSL, EDL, LDL, and the soil. Different letters indicate a significant difference (*P* < .05).

The Shannon index of decomposer bacteria in both the early-stage and the late-stage were significantly higher than the phyllosphere bacteria for both ash and pine, and the Shannon index of bacteria in the late-stage decomposing litter was the highest (Fig. 2A). The results of principal coordinate analysis (PCoA) and permutational multivariate analysis of variance (PERMANOVA) revealed significant differences (*P* < .05) in the bacterial community composition between the phyllosphere and the decomposing ash litter (both early-stage and late-stage, Fig. 2B, Table S2). In contrast, for pine litter, bacterial communities in the early-stage decomposing litter were not significantly different from those in the phyllosphere and soil (*P* > .05). Additionally, there was no significant difference in bacterial communities between the late-stage decomposing litter and soil. However, a significant difference in bacterial communities was observed between the phyllosphere and the late-stage decomposing litter (*P* < .05, Fig. 2B, Table S2). The fungal Shannon index of the early-stage decomposing ash litter exhibited a similar trend (Fig. 3A), but in the late-stage decomposing litter, the Shannon index did not differ significantly from that of the early-stage decomposing litter and soil (*P* > .05). The Shannon index of fungi in the decomposing (both early-stage and late-stage) litter of pine was not significantly different from that of the phyllosphere (*P* > .05). But the Shannon index of fungi in the late-stage decomposing litter was lower than that for fungi in soil significantly (*P* < .05, Fig. 3A). The PCoA and PERMANOVA results indicated that, for ash, the composition of the fungal community was significantly different between the phyllosphere and the decomposing litter (both early-stage and late-stage, *P* < .05), but no significant difference (*P* > .05) was observed between the late-stage and soil (Fig. 3B, Table S2). In the case of pine, no significant difference was found between the phyllosphere and the early-stage decomposing litter (*P* > .05). However, there was a significant difference in fungal communities present between the early-stage and the late-stage decomposing litter (*P* < .05), while no significant difference (*P* > .05) was found between the late-stage decomposing litter and soil (Fig. 3B, Table S2).

### Key microorganisms in decomposing litter

The relative abundance of *Alphaproteobacteria* (including the genera *Brevundimonas*, *Aureimonas_A_501427* and *Sphingomonas_L_486704*), *Gammaproteobacteria* (including the genera *Variovorax*, *Achromobacter* and *Pseudomonas_E_647464*), *Actinomycetia* (including the genus *Frigoribacterium*) and *Bacteroidia* (including the genus *Flavobacterium*) was the top four dominant bacterial classes in the decomposing litter (Fig. 2C, Fig. S1). For ash, the relative abundance of *Alphaproteobacteria* (including the genus *Sphingomonas_L_486704*) gradually decreased, while *Bacteroidia* (including the genus *Flavobacterium*) gradually increased from the phyllosphere to the early-stage decomposing litter and then to the late-stage decomposing litter. In contrast, the relative abundances of both *Alphaproteobacteria* and *Bacteroidia* (including the genus *Flavobacterium*) increased progressively, whereas *Gammaproteobacteria* (including the genus *Achromobacter*) showed a decreasing trend with decomposing stage in pine litter (Fig. 2C, Fig. S1). For fungi, the dominant classes identified in decomposing litter were *Dothideomycetes* (including the genera *Saccharata*, *Cercospora*, and *Fusicladium*), *Leotiomycetes* (including the genera *Pezicula*, *Xenopolyscytalum*, and *Phialea*), *Sordariomycetes* (including the genus *Trichoderma*), and *Agaricomycetes*. The relative abundance of *Dothideomycetes* (including the genus *Cercospora*) showed a decreasing trend, while *Sordariomycetes* (including the genus *Trichoderma*) gradually increased from the phyllosphere to the early-stage decomposing litter and then to the late-stage decomposing litter for both ash and pine (Fig. 3C, Fig. S1).

The top 400 high relative abundance ASVs (Amplicon Sequence Variant) were selected for network analysis in the decomposing litter. The findings revealed that the number of the late-stage decomposing litter network edges was significantly higher than that of the early-stage decomposing litter network edges (Fig. S2). In addition, we selected key species from the bacterial network associated with the early-stage decomposing litter, and 59.5% and 58.3% of ASVs were detected in the phyllosphere, while 27.0% and 19.4% were detected in the soil of ash and pine, respectively (Fig. S2, Supplementary Data 1). In contrast, within the late-stage network, 17.5% and 17.5% of ASVs were detected in the phyllosphere, whereas a significantly larger proportion, 70.0% and 67.5% were found in the soil (Fig. S2, Supplementary Data 1). For the fungal network, 17.1% and 41.7% of ASVs were present in the phyllosphere, and 48.6% and 19.4% were in the soil during the early-stage. However, during the late-stage, 17.5% and 0% of ASVs were observed in the phyllosphere, with 60.0% and 51.3% being detected in the soil (Fig. S2, Supplementary Data 2).

### Assembly process of litter decomposition microorganisms

**Figure 4:**
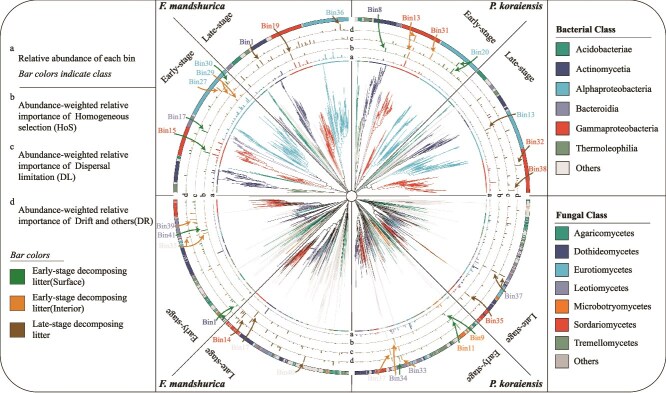
Variations of assembly processes across different phylogenetic groups of bacteria and fungi during the litter decomposition process. Phylogenetic tree was displayed at the center. a: Relative abundance of each bin. Bar colors indicate class. b, c, d: Abundance-weighted relative importance of homogenous selection (HoS), dispersal limitation (DL), drift and others (DR). Bar colors indicate different niches. Detailed information is provided in Supplementary Data 3 and Supplementary Data 4.

**Figure 5:**
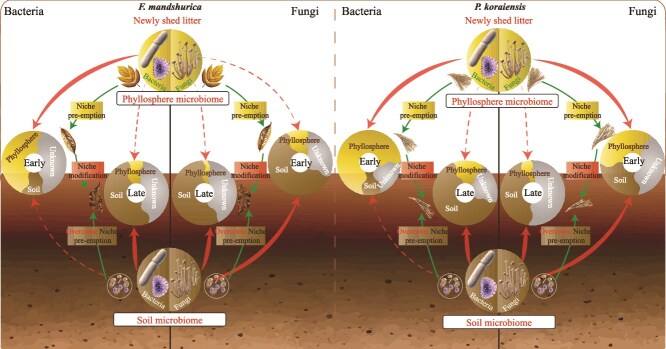
A simplified conceptual model presenting the origin and succession processes of microbes responsible for litter decomposition. The red arrows represent the respective contributions of phyllosphere microorganisms and soil microorganisms to the early and late stages of litter decomposition. Dotted lines indicate lesser contributions, while solid lines signify greater contributions. The green arrows represent the ecological processes driving microbial succession, transitioning from the newly shed litter to the early and late stages decomposing litter.

The microbial community composition in the litter showed a significant correlation with the litter’s intrinsic properties such as litter quality and enzyme activities, especially for ash (Fig. S3, Table S3, Table S4). These physicochemical characteristics established distinct habitat conditions, thereby shaping the assembly of litter microbial communities. Dispersal limitation, drift, and homogeneous selection were more important than other processes in microbial community assembly in the decomposing litter (Fig. 4, Fig. S4). For bacteria, homogeneous selection was the primary assembly process for both ash and pine communities. But drift emerged as the predominant process of fungal community assembly (Fig. 5, Fig. S4). As decomposition progressed from the early-stage to the late-stage, the relative importance of deterministic processes (heterogeneous selection and homogeneous selection) in bacterial assembly decreased significantly. Specifically, for ash litter, these processes declined from 63.7% to 46.2%, while for pine litter, they dropped from 52.7% to 32.1%. In contrast, stochastic processes (dispersal limitation, homogenizing dispersal, and drift) governed fungal community assembly for both ash and pine communities (Fig. S4).

Based on phylogenetic-bin-based null model (iCAMP), ASVs with an abundance greater than 100 reads were selected in the bacterial and fungal communities, which were subsequently categorized into varied bin numbers. By counting the bacterial abundance of each bin, the impact of homogeneous selection was primarily attributed to the responses of bins within *Gammaproteobacteria* (both early-stage and late-stage) and *Alphaproteobacteria* (early-stage) (Fig. 4, Supplementary Data 3). For pine, the impact of homogeneous selection was mainly attributed to the response of bins in *Actinobacteria* and *Gammaproteobacteria* in the early-stage decomposing litter and *Alphaproteobacteria* in the late-stage decomposing litter (Fig. 4, Supplementary Data 3). In the case of ash fungi, the most abundant bins governed by dispersal limitation were from *Dothideomycetes* (early-stage), *Leotiomycetes* (early-stage), and *Sordariomycetes* (late-stage) (Fig. 4, Supplementary Data 4). For fungi of pine litter, the most abundant bins governed by dispersal limitation were from *Microbotryomycetes* (early-stage) and *Leotiomycetes* (both early-stage and late-stage) (Fig. 4, Supplementary Data 4).

## Discussion

### The origins of the microbes responsible for litter decomposition

Inconsistent with our hypothesis 1, the origins of microbes in the decomposing litter were not always dominated by microbes from the soil. In the early-stage decomposing pine litter, phyllosphere bacteria and fungi were the main sources. The network analysis and relative abundance succession results revealed that *Gammaproteobacteria* (including genera such as *Pseudomonas*, *Caballeronia*, *Robbsia,* and *Polaromonas*) and *Actinomycetia* (including the genus *Nakamurella*) were the dominant bacterial classes among pine litter microorganisms originating from the phyllosphere (Fig. 2C, Fig. S2 and Supplementary Data 1). *Leotiomycetes* (represented by *Lophodermium*), *Tremellomycetes*, *Dothideomycetes* (represented by *Paradevriesia*), *Sordariomycetes* (represented by *Nigrospora*), and *Microbotryomycetes* (represented by *Colacogloea*) were the predominant fungal classes in pine litter microorganisms, derived from the phyllosphere (Fig. 3C, Fig. S2 and Supplementary Data 2). In the early-stage decomposing ash litter, fungi primarily originated from the soil while bacteria were mainly from the phyllosphere (Fig. 1). The dominant bacterial classes from phyllosphere were *Actinomycetia* (e.g. *Amnibacterium*), *Alphaproteobacteria* (e.g. *Brevundimonas* and *Rhizobium*), *Bacteroidia* (e.g. *Chryseobacterium*), and *Gammaproteobacteria* (e.g. *Duganella*, *Massilia* and *Pseudomonas*) in ash litter (Fig. S2, Supplementary Data 1). *Leotiomycetes* (represented by *Pezicula*), *Microbotryomycetes*, *Dothideomycetes* (represented by *Paraphaeosphaeria*), *Sordariomycetes* (represented by *Trichoderma* and *Cosmospora*), *Rozellomycotina_cls_Incertae_sedis,* and *Eurotiomycetes* (represented by *Penicillium*) were the predominant fungal classes among ash litter microorganisms, which originated from the soil (Fig. S2, Supplementary Data 2). Previous studies have highlighted the significant role of these microorganisms in litter decomposition [14, 16]. Notably, *Phialea* (Class *Lophodermium*) has frequently been observed in pine litter compared to ash litter [35, 36], suggesting that low-quality litter may necessitate more specialist microorganisms for decomposition.

For the late-stage decomposing litter, both ash and pine litter exhibited a dominant soil source of microbes, although a significant proportion of unknown sources remained in the pine litter fungi, which could be from air or invertebrates (e.g. insects, earthworms) carrying microbial propagules (Fig. 1). This could also be due to the incomplete characterization of diversity, particularly the presence of rare species, within the defined sources. It is worth mentioning that *Bionectria* (class *Sordariomycetes*), a key microorganism from the soil, had been consistently present in pine litter throughout both the early and late stages of decomposition, contributing to the breakdown of organic matter [37]. Similarly, saprophytic fungi such as *Pezicula* (class *Leotiomycetes*, which degrades complex organic matter [38]) from the soil and *Pseudomonas* (class *Gammaproteobacteria*, degrades simple organic matter [39]) from the phyllosphere had been consistently present and played a crucial role in the decomposition of ash litter. The diversity of phyllosphere interior bacteria was the lowest among all bacteria groups (Fig. 2) and whether phyllosphere microbes can remain during decomposition did not depend on their diversity. We further explore the mechanisms of microbial succession during the decomposition process.

### The assembly processes of the litter decomposing microbes

Slightly different from our hypothesis 2, our study revealed that phyllosphere bacteria and soil fungi occupied specific niches during the early stage of litter decomposition (Fig. 1 and 5). Hence, we believe that the niche pre-emption effect was co-regulated by resource-based niches and dispersal limitation, which refers to uniform selective pressures that shape the microbial community composition in a similar way in the litter during the succession process due to the consistent environmental conditions and resource availability [40]. Our results show that homogeneous selection and dispersal limitation were the primary processes governing microbial assembly during the early-stage decomposing litter (Fig. S4). When leaves fell, the phyllosphere microorganisms that had been consistently present on the leaves dominate (homogeneous selection). Once the leaves came into contact with the ground, microorganisms from the surrounding habitat attempt to colonize the litter. However, due to differences in niches between the surrounding habitat and the litter, dispersal limitation occurred. Although bacteria are smaller and capable of moving faster, their mobility often needs carriers [41]. For example, fungi were found to serve as “highways” for particular bacterial dispersal from soil [41, 42]. Fungi mycelium can also utilize nutrient sources more efficiently than bacteria [43]. In addition, fungal communities may have a broader niche width and greater functional diversity compared to bacteria [44], and may be less susceptible to disturbance [45]. The succession of the fungal community was mainly dominated by stochastic processes including dispersal limitation and drift (Fig. S4). However, even in the presence of dispersal limitations, some fungi (*Ascomycota* and *Basidiomycota*) can still bypass these restrictions by spores and hyphae, thereby colonizing the litter. Once the dispersal barrier was overcome, phyllosphere fungi were not able to compete with soil fungi because they have similar resource-based niches and soil fungal biomass was higher (mass effect). Therefore, phyllosphere bacteria (who mainly use simple organic compounds [46]) had different resource-based niches as compared to soil fungi (who mainly use cellulose and lignin [14]), and co-existed in the decomposing litter. The drift process refers to the random changes in the composition and abundance of microbial species in the litter due to stochastic events rather than deterministic (selection-driven) factors [40] and it explained 25% of the microbial (both bacteria and fungi) assembly process (Fig. 4, Fig. S4). As time went on, drift process got weaker and dispersal limitation process got stronger for fungal succession, suggesting that dominant decomposers stabilized community composition, which has been reported before [16, 17].

Over time, with some compounds consumed during litter decomposition, the ecological niches were modified. It has been reported that different litter types and microbial colonization could create different niches as litter decomposed [47, 48]. Therefore, we believe niche modification mechanism prevailed the succession during the late decomposition stage, which both soil bacteria and soil fungi were able to colonize the decomposing litter, which were affected by the drift and dispersal limitation processes (Fig. S4). Still, due to the less niche overlap with phyllosphere bacteria (Fig. S5) for soil fungi, they occupied the decomposing litter more than soil bacteria, suggesting the importance of fungi in litter decomposition in forest ecosystems.

### The important role of litter quality in microbial succession during litter decomposition

Consistent with our hypothesis 3, we found that the microbial succession processes differed between pine and ash litter, mainly due to their differences in chemical composition (Fig. 5). Ash litter had a lower C/N ratio and more abundant resources (Table S3), creating more favorable conditions for rapid multiplication of phyllosphere bacteria than pine litter. Thus, the easily accessible substances could be more rapidly consumed by phyllosphere bacteria [6] in ash litter than in pine litter, also modifying the ecological niche for microbes more rapidly. In contrast, pine litter was characterized by a higher C/N ratio and more limited resources than ash litter (Table S4), making its degradation highly energy-intensive and enzyme dependent [49]. In addition, the small leaf area, and the presence of specific volatile compounds in pine needles [50] could also block the invasion of soil microbes, even for fungi with high ability of overcoming dispersal limitation. The lower decomposition rate meant fewer changes in ecological niches on the same time scale for pine than for ash litter, therefore indicating less niche modification for microbial succession. The microbial community composition was less correlated with the quality of the litter and the activities of hydrolytic and oxidative enzymes in the pine litter than in the ash litter (Fig. S3), suggesting that low-quality pine litter may require specific microbial groups (e.g. *Phialea* [Class *Lophodermium*]) for decomposition.

Overall, we agree that phyllosphere and soil microbes collaboratively contributed to HFA through complementary mechanisms [13]. The phyllosphere niche pre-emption effect is likely weaker at home than at foreign environment because native soil decomposers have higher ability to outcompete phyllosphere microbes, resulting in higher decomposition rate at home, which is supported by the higher decomposition rate of ash litter with weaker niche pre-emption effects in our study (Table S3). This is different from previous findings of stronger niche pre-emption effect at home [13] because we found phyllosphere bacteria mediated initial decomposing when soil fungi colonized the litter, but gradually lost their dominance as decomposition progressed. Instead, the phyllosphere niche modification effect likely resulted in higher HFA because it created an environment fine-tuned to home-field decomposers but potentially suboptimal for foreign decomposers, which has been reported before [13]. Therefore, we believe that phyllosphere microbes play a major role in HFA by precolonizing leaves and influencing microbial succession and this effect is stronger for low-quality litter such as pine litter as compared to high-quality litter.

In conclusion, the microbiome in the early-stage decomposing litter came from phyllosphere bacteria and soil fungi, while bacteria and fungi in the late-stage decomposing litter were from the soil primarily. Overall, the keystone taxa involved in litter decomposition were the bacterial classes *Actinomycetia*, *Alphaproteobacteria*, *Bacteroidia*, and *Gammaproteobacteria*, along with the fungal classes *Dothideomycetes*, *Leotiomycetes*, and *Sordariomycetes*. However, the specific assembly progress in litter had an important relationship with litter quality. Our research has elucidated the origin and assembly processes of microorganisms involved in litter decomposition, highlighting the pivotal role of microbial succession in driving litter decomposition process and indirectly explaining HFA. These findings established a foundation for linking microbial functions to global biogeochemical cycles and incorporating microbial succession into land management to enhance soil health and carbon sequestration. Tracking microbial shifts can improve predictions of climate change impacts on litter decomposition. A limitation of our study is the need for greater taxonomic resolution to elucidate the specific roles of diverse microbial species in decomposition, as well as the necessity of culture-based experiments to verify the accuracy of our functional predictions.

## Supplementary Material

Supplementary_Data_1_ycaf155

Supplementary_Data_2_ycaf155

Supplementary_Data_3_ycaf155

Supplementary_Data_4_ycaf155

Supplementary_Information_ycaf155

## Data Availability

The sequence data for both bacterial and fungal communities have been submitted to the NCBI Sequence Read Archive (SRA), under the Bioproject number PRJNA1211192.
