# Supplementary material for: The origin and succession of the microbial community in decomposing litter
Source: ISME Commun. 2025 Sep 5;5(1):ycaf155. doi: 10.1093/ismeco/ycaf155 (PMC12477604; doi:10.1093/ismeco/ycaf155)
Supplement: Supplementary_Information_ycaf155 [file supplementary_information_ycaf155.pdf]

Table S1 PERMANOVA results testing the effect of altitude on β-diversity of bacterial and fungal communities in different Plant species

	Plant species	F values	*P* values
Bacteria	*F. mandshurica*	2.09	> 0.05
	P. koraiensis	2.01	> 0.05
Fungi	*F. mandshurica*	2.47	> 0.05
	P. koraiensis	1.91	> 0.05

Table S2 Pairwise Permutational Multivariate Analysis of Variance (PERMANOVA) results for bacterial and fungal β-diversity in decomposing litter and soil.

The comparisons include NSL (newly shed litter), EDL (early-stage decomposing litter), LDL (late-stage decomposing litter), and soil samples from different periods (early and late). *P*-values < 0.05 indicate the statistical significance of group-level differences.

	Plant species	Pairs	*P* values
Bacteria	*F. mandshurica*	NSL vs EDL	< 0.05
		NSL vs LDL	< 0.05
		EDL vs LDL	< 0.05
		EDL vs Soil (early)	< 0.05
		LDL vs Soil (late)	< 0.05
	P. koraiensis	NSL vs EDL	> 0.05
		NSL vs LDL	< 0.05
		EDL vs LDL	< 0.05
		EDL vs Soil (early)	> 0.05
		LDL vs Soil (late)	> 0.05
Fungi	*F. mandshurica*	NSL vs EDL	< 0.05
		NSL vs LDL	< 0.05
		EDL vs LDL	< 0.05
		EDL vs Soil (early)	< 0.05
		LDL vs Soil (late)	> 0.05
	P. koraiensis	NSL vs EDL	> 0.05
		NSL vs LDL	< 0.05
		EDL vs LDL	< 0.05
		EDL vs Soil (early)	> 0.05
		LDL vs Soil (late)	> 0.05

Table S3 Differences in the properties of the newly shed litter and decomposing litter (early-stage and late-stage) of *F. mandshurica*

One-way analysis of variance was conducted to compare the litter quality, as well as oxidase and hydrolase activities in *F. mandshurica*, between three distinct phases: newly shed litter, early-stage decomposing litter and late-stage decomposing litter. The data are presented as mean ± standard deviations. Different letters indicate significant difference between different phases at $P < 0.05$.

	Newly shed litter	Early-stage decomposing litter	Late-stage decomposing litter
Total Carbon (g kg^{-1})	454.67±6.28b	466.88±8.17a	458.68±12.83b
Total Nitrogen (g kg^{-1})	11.29±1.97c	19.92±2.97b	25.64±2.88a
C/N	41.45±7.38a	23.97±3.86b	18.16±2.64c
Cellulose (%)	22.49±2.43a	19.36±2.80b	15.59±2.84c
Lignin (%)	18.40±4.75c	35.62±2.95b	43.79±3.04a
β-1,4-Glucosidase (nmol g^{-1} h^{-1})	172.68±90.47c	1137.71±189.76b	1547.76±184.59a
β-Xylosidase (nmol g^{-1} h^{-1})	5.12±2.97c	57.24±13.70b	123.94±32.08a
β-cellobiohydrolase (nmol g^{-1} h^{-1})	21.28±17.84c	361.93±95.15b	715.99±106.98a
Polyphenol oxidase (nmol g^{-1} h^{-1})	0.45±0.21c	1.63±0.87b	3.08±0.45a
Peroxidase (nmol g^{-1} h^{-1})	0.41±0.22c	1.68±0.71b	3.92±0.41a

Table S4 Differences in the properties and hydrolase activities of newly shed litter and decomposing litter (early-stage and late-stage) of *P. koraiensis*.

The data are presented as mean ± standard deviations. Different letters indicate significant difference between different phases at $P < 0.05$.

	Newly shed litter	Early-stage decomposing litter	Late-stage decomposing litter
Total Carbon (g kg^{-1})	523.33±8.12a	522.73±9.36a	494.68±10.56b
Total Nitrogen (g kg^{-1})	4.52±0.66c	7.31±2.27b	16.65±3.15a
C/N	117.65±14.64a	77.82±22.51b	30.66±5.47c
Cellulose (%)	6.11±3.67c	9.22±3.04b	18.21±1.53a
Lignin (%)	65.55±3.60a	60.57±4.57b	50.23±4.70c
β-1,4-Glucosidase (nmol g^{-1} h^{-1})	373.92±72.70c	545.77±85.10b	1120.26±295.47a
β-Xylosidase (nmol g^{-1} h^{-1})	32.44±10.67b	38.13±12.72b	71.18±30.68a
β-cellobiohydrolase (nmol g^{-1} h^{-1})	172.94±35.00b	182.11±62.88b	316.61±88.36a
Polyphenol oxidase (nmol g^{-1} h^{-1})	0.09±0.03c	0.41±0.21b	1.98±0.44a
Peroxidase (nmol g^{-1} h^{-1})	0.09±0.04b	0.36±0.09b	2.87±0.88a

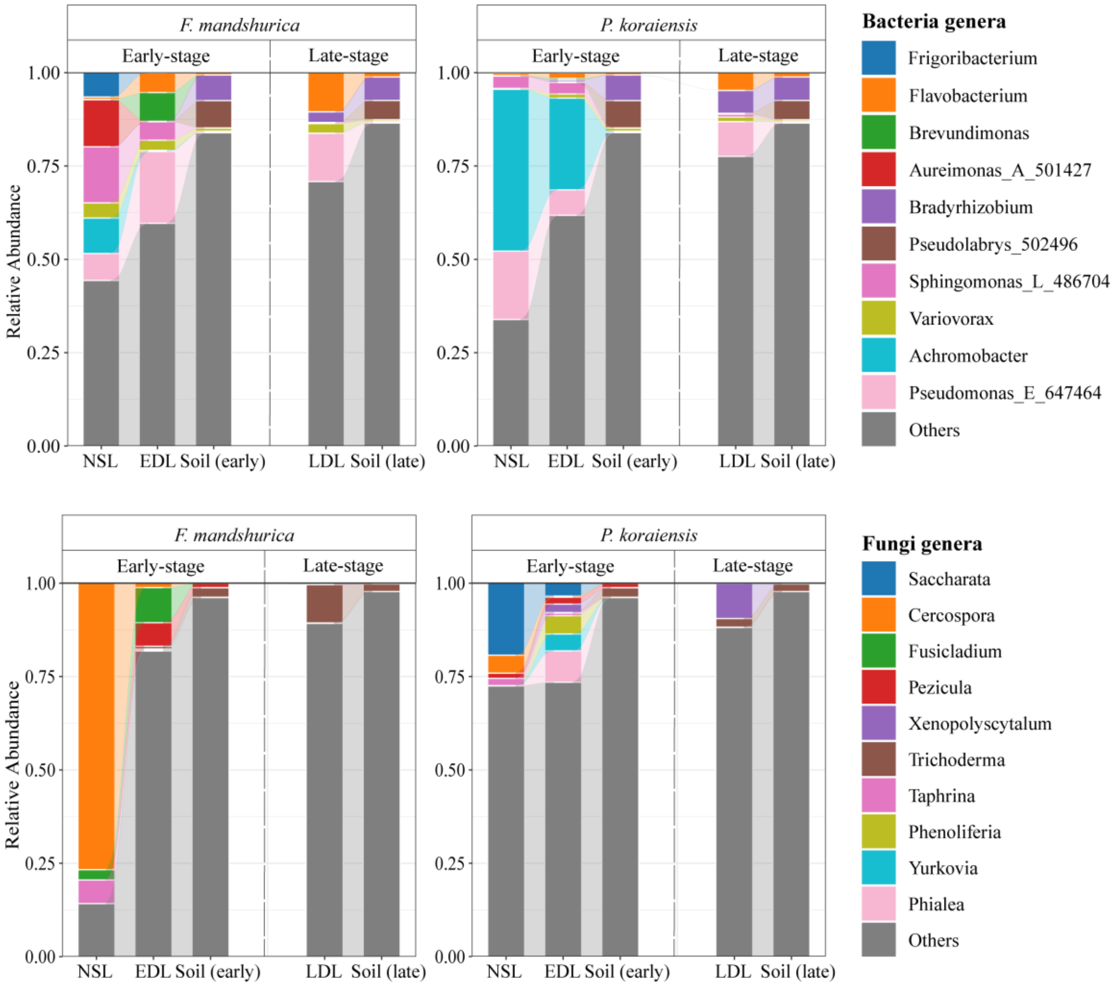

Figure S1 Relative abundance of the bacteria and fungi (genus) in the newly shed litter (NSL), the early-stage decomposing litter (EDL), the late-stage decomposing litter (LDL) and the soil.

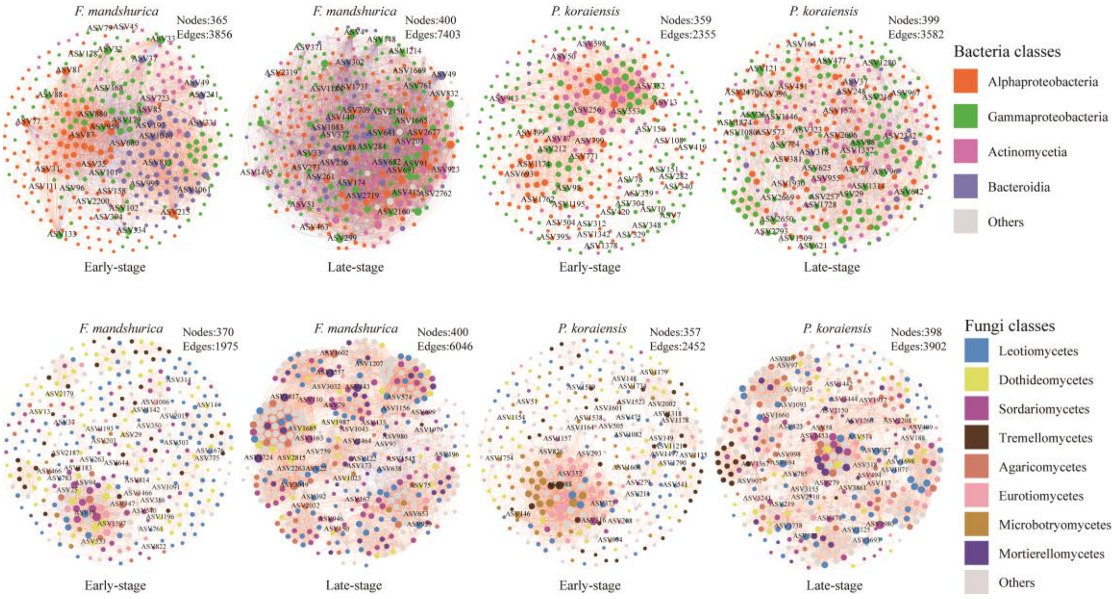

Figure S2 Co-occurring network of the bacteria and fungi in the early-stage decomposing litter and late-stage decomposing litter.

The nodes are colored according to classes. Node size is proportional to the degree centrality of each ASV. The red line represents a positive correlation, while the blue line represents a negative correlation. The nodes are labeled with the keystone species associated with the early-stage and late-stage decomposing litter; detailed information is provided in Supplementary Data 1 and Supplementary Data 2.

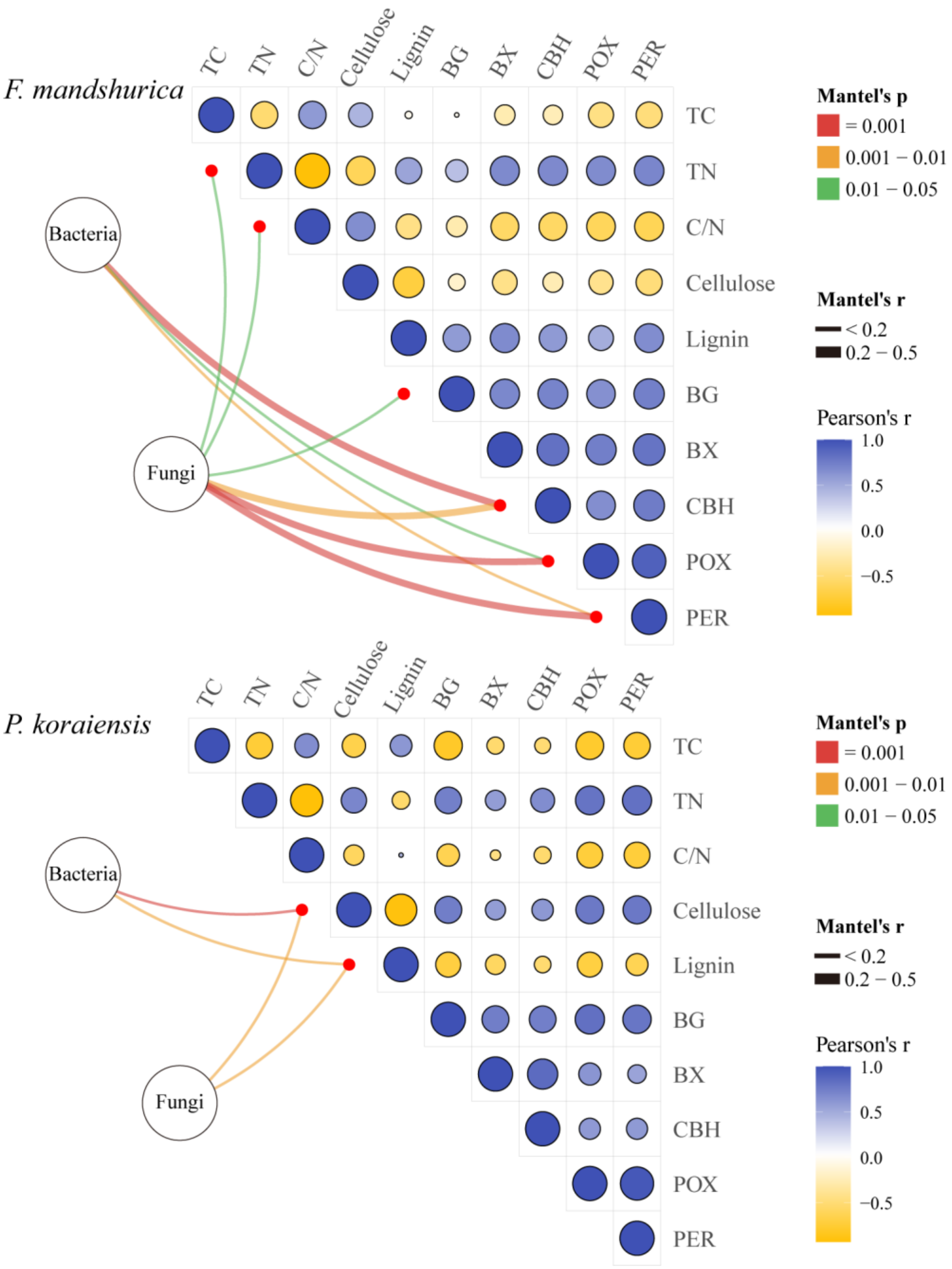

Figure S3 Correlations between microbial community composition (Genus-level composition of microorganisms and their relative abundance) and litter properties in the decomposing litter of two plant species by Mantel analysis.

Bacterial and fungal community composition in relation to litter properties of *F. mandshurica* and *P. koraiensis*. TC: content of total carbon, TN: content of total nitrogen, C/N: (total carbon) / (total nitrogen), BG: β-1,4-Glucosidase activity, BX: β-Xylosidase activity, CBH: β-cellobiohydrolase activity, POX: phenol oxidase activity, PER: peroxidase activity.

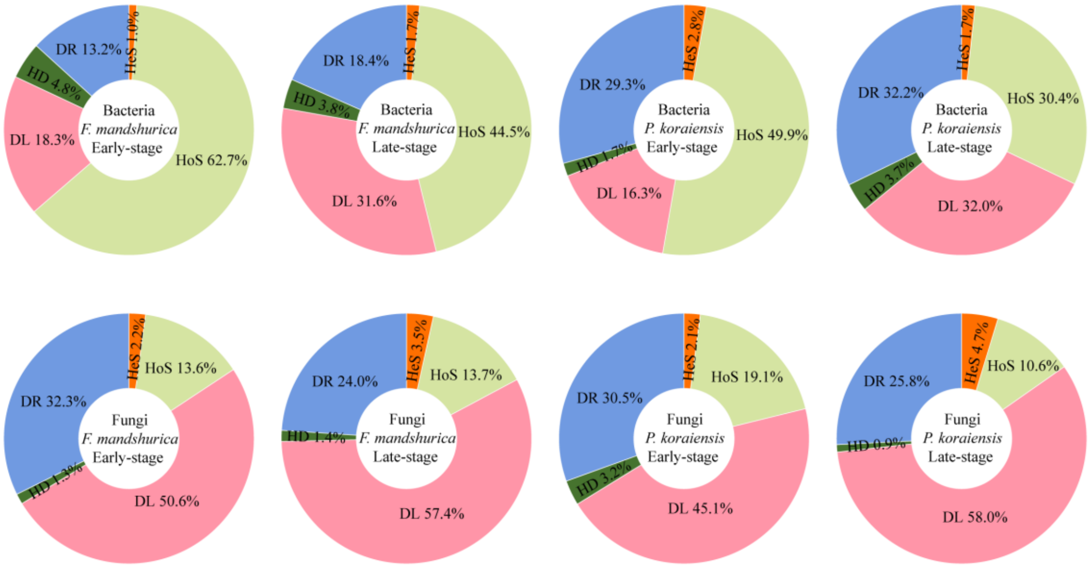

Figure S4 Relative importance of different assembly processes in the partly-decomposed litter of two plant species.

HoS: Homogeneous selection, HeS: Heterogeneous selection, DL: Dispersal limitation, HD: homogenizing dispersal, DR: drift (and others).

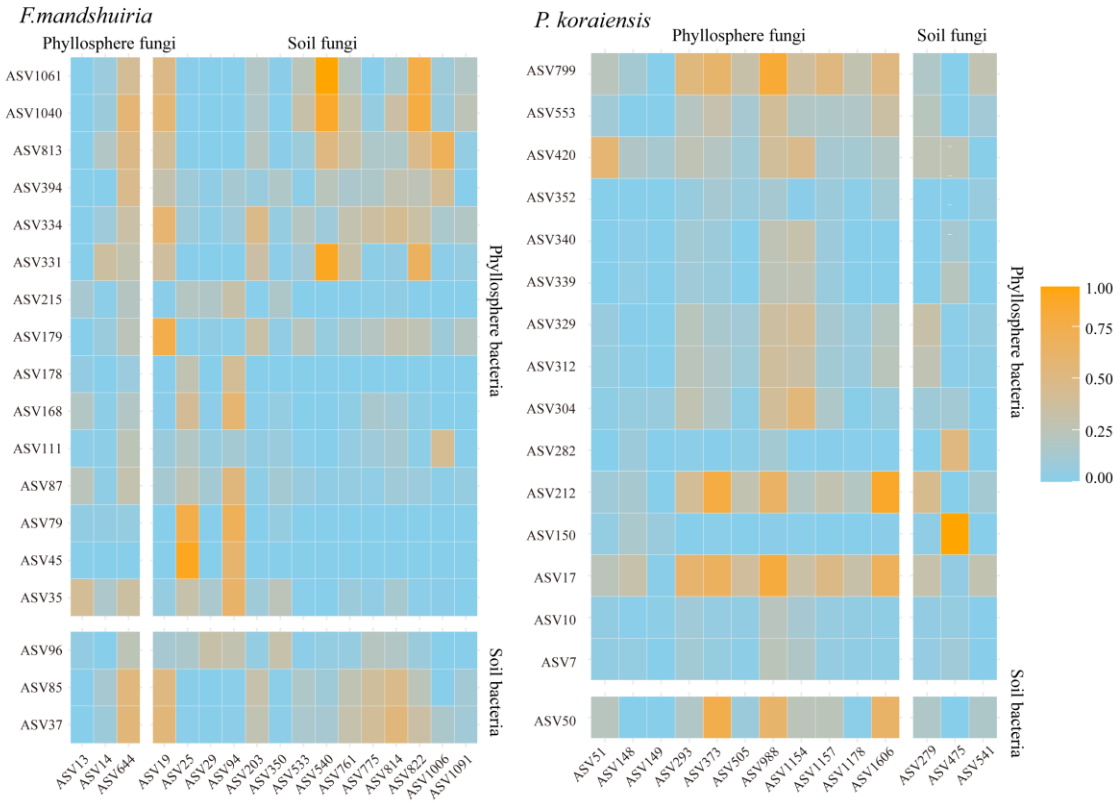

Figure S5 Niche overlap relationships of keystone species in the early-stage decomposing litter of *F. mandshurica* and *P. koraiensis*

Species selection is based on a single source—either phyllosphere or soil. A niche overlap index of 0 indicates no overlap, while an index of 1 represents complete overlap.
